# Preparation of Long Sisal Fiber-Reinforced Polylactic Acid Biocomposites with Highly Improved Mechanical Performance

**DOI:** 10.3390/polym13071124

**Published:** 2021-04-02

**Authors:** Zhifang Liang, Hongwu Wu, Ruipu Liu, Caiquan Wu

**Affiliations:** 1 Guangdong Provincial Key Laboratory of Technique and Equipment for Macromolecular Advanced Manufacturing, South China University of Technology, Guangzhou 510640, China; mezhifangl@mail.scut.edu.cn (Z.L.); 201821003313@mail.scut.edu.cn (R.L.); 2 Key Laboratory of Polymer Processing Engineering, South China University of Technology, Ministry of Education, Guangzhou 510640, China; 201320100894@mail.scut.edu.cn; 3 National Engineering Research Center of Novel Equipment for Polymer Processing, South China University of Technology, Guangzhou 510640, China

**Keywords:** PLA, sisal fiber, continuous long fiber-reinforced composites, mechanical properties

## Abstract

Green biodegradable plastics have come into focus as an alternative to restricted plastic products. In this paper, continuous long sisal fiber (SF)/polylactic acid (PLA) premixes were prepared by an extrusion-rolling blending process, and then unidirectional continuous long sisal fiber-reinforced PLA composites (LSFCs) were prepared by compression molding to explore the effect of long fiber on the mechanical properties of sisal fiber-reinforced composites. As a comparison, random short sisal fiber-reinforced PLA composites (SSFCs) were prepared by open milling and molding. The experimental results show that continuous long sisal fiber/PLA premixes could be successfully obtained from this pre-blending process. It was found that the presence of long sisal fibers could greatly improve the tensile strength of LSFC material along the fiber extension direction and slightly increase its tensile elongation. Continuous long fibers in LSFCs could greatly participate in supporting the load applied to the composite material. However, when comparing the mechanical properties of the two composite materials, the poor compatibility between the fiber and the matrix made fiber’s reinforcement effect not well reflected in SSFCs. Similarly, the flexural performance and impact performance of LSFCs had been improved considerably versus SSFCs.

## 1. Introduction

With the increasing emphasis on environmental protection and resource conservation in recent years, there has been great interest in the preparation of green biocomposites using sustainable resources or degradable materials. In the field of composite materials, biobased and biodegradable resins, instead of petroleum-based resins, have been gradually used to prepare fully biodegradable composites from high-quality and low-priced natural plant fibers [1]. A large number of biodegradable polymer matrices, such as polysaccharide derivatives and polyesters, and a great quantity of plant fibers such as kenaf, jute, sisal, nanocellulose and microfibrillarized cellulose are gradually used in biocomposites [2]. Biocomposites with higher biobased content made of plant fibers and crop-based plastics have also been developed continuously [3], which is meaningful for alleviating oil resource tension and achieving the sustainable development of resources.

Polylactic acid (PLA), one of the most widely used biobased and biodegradable polymers, can be prepared from a wide range of raw materials, especially starch-rich crops such as sugar beet, corn and wheat [2]. It can be produced by the direct polycondensation of lactic acid obtained from starch fermentation, or by the ring-opening polymerization of lactide, which is a common synthetic method for the industrial production of PLA [4,5,6]. The attractive physical properties, mechanical properties, biodegradability and biocompatibility of PLA make it a very promising material in industrial applications [7,8]. However, compared with other commonly used petroleum-based polymers, the high cost and poor toughness of PLA limit its widespread use [9]. The development of viable green technologies aimed at enhancing biodegradable and biocompatible aliphatic polyesters such as PLA has aroused great interest [10]. In order to maintain the biodegradability of products, natural plant fibers from the same wide range of sources are usually used to make all biobased composites with PLA, which not only reduces costs but also improves the overall performance of products [11].

Plant fiber is extracted from plants grown in nature, and it is widely distributed in all parts of plants. It has the advantages of low cost, light weight and high mechanical strength, and is renewable in nature, making it a green resource in line with sustainable development. Sisal fiber (SF) is a natural fiber crop of the agave family, mainly grown in tropical and subtropical regions. It is composed of cellulose, some hemicellulose and a small amount of lignin. Sisal fiber has relatively high fiber strength and long fibers, compared with other plant fibers [12]. The presence of hydroxyl groups in SF leads to poor compatibility between SF and hydrophobic polymer matrices [3]. Physical and chemical methods are often used to improve the interface properties of plant-fiber-reinforced resin composites [13]. Ott et al. [14] treated jute fiber with alkali to obtain good adhesion between the fiber and matrix. 

Due to poor interface bonding properties and the inherent defects of plant fibers, it is difficult to achieve the desired level of performance improvement in plant-fiber-reinforced composite materials at present. Researchers have been studying the improvement of the pretreatment process for fibers and polymers, mainly focusing on the use of more effective surface modification technology to improve the interface bonding performance between the fiber and the matrix [15,16,17,18,19]. The kind, composition, form and concentration of fiber fillers are all involved in determining the properties of fiber-reinforced composites. Generally, natural-fiber-reinforced plastic composite materials are manufactured using traditional manufacturing techniques, such as injection molding, extrusion molding and compression molding. Although the addition of plant fibers in resin has certain advantages in reducing costs and improving performance, the characteristics of plant fibers make it difficult to blend well with polymer matrices, especially in continuous processing like extrusion. They are often cut into short ones, or milled for feeding. In addition, strong melt blending will reduce the aspect ratio of plant fibers [20]. Saurabh Chaitanya [9] developed short sisal fiber-reinforced PLA biocomposites using injection molding. It was found that the strength of the composites did not improve well due to poor fiber dispersion. The aspect ratio of fibers has an important influence on the characteristics of biocomposites. Processing technology should be able to maintain a high fiber aspect ratio and uniform dispersion in the developed biocomposites [21]. Continuous long fibers could help to promote homogeneous stress and strain fields, which play a key role in the improvement of mechanical properties [22]. Shinji Ochi [23] prepared long fiber premixes by placing kenaf fiber into a PLA emulsion and drying it. Then unidirectional long-fiber-reinforced composites were fabricated by hot-pressing. This is a solution method for premixing.

In this paper, a facile and economical extrusion-rolling melt blending method was applied to prepare continuous long sisal fiber/PLA premixes, and then they were molded to obtain biocomposite materials. Compared to traditional blending methods, this kind of unidirectional continuous long sisal fiber-reinforced PLA composites benefited from enhanced interface adhesion between the fiber and the PLA matrix, and good dispersion of dispersed phases, attributed to the fact that the long fiber premix was produced through rolling compression. The SF remained a continuous long length in the composites. The effect of long sisal fiber on the mechanical properties of the composite materials was studied. For comparison, random short sisal fiber/PLA composites were prepared through open milling and compression molding. The results demonstrate that this processing technology is a prospective method for plant-fiber-reinforced polymer composites manufacture.

## 2. Materials and Methods

### 2.1. Materials

Sisal fiber was purchased from Dongfang Sisal Group Co. (Guangdong, China). The length of high-quality sisal fibers was at least 95 cm, and the moisture regain of the fiber was not more than 13%. The sisal fiber has a tensile strength of about 500 MPa and an elongation at break of 2.0~2.5% [24]. Injection grade PLA 3051D, manufactured by American Nature Works, had a specific gravity of 1.24 g/cm^3^ and a melt flow rate of 14 g/10 min (210 °C, 2.16 kg). Sodium hydroxide with analytical purity was purchased from Nanjing Chemical Reagent Co. (Jiangsu, China).

### 2.2. Fiber Treatment

**Figure 1:**
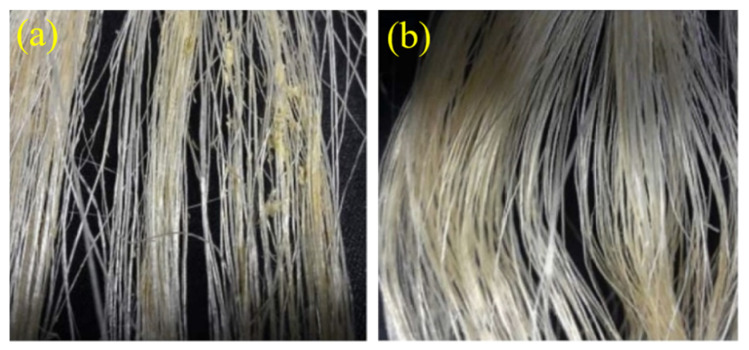
Sisal fibers (**a**) before and (**b**) after alkali treatment.

Prior to blending with PLA, sisal fibers were subjected to alkali treatment. While washing with sodium hydroxide, some small molecular weight impurities such as wax, pectin and part of the lignin and hemicellulose in the fibers were dissolved and removed [13,25,26]. Meanwhile, the alkali treatment reduced the fiber diameter and thus increased the aspect ratio of the fibers [27]. This increased the effective fiber surface area and facilitated interface adhesion between the fiber and the matrix [28]. For this treatment, the long fiber raw materials were immersed in a prepared 2 wt% NaOH aqueous solution for 40 min at room temperature. After that, the fibers were taken out and washed with distilled water several times until the solution was neutral. Then the treated fibers were placed in an oven and dried at 80 °C for 12 h for later use. Figure 1 shows pictures of the sisal fiber before and after alkali treatment.

### 2.3. Premixes Preparation

#### 2.3.1. Preparation of Continuous Long Sisal Fiber/PLA Premixes 

**Figure 2:**
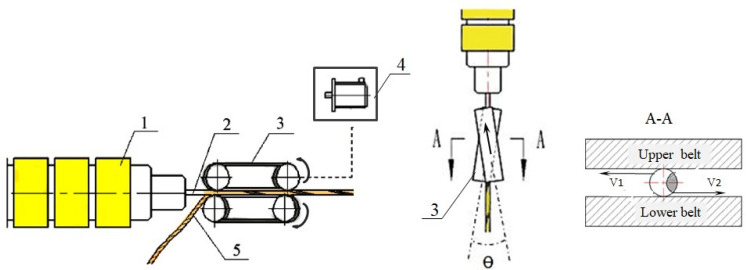
Structure diagram of online extrusion-rolling blending system(1 Extruder; 2 PLA extrudates; 3 Conveyor belts; 4 Drive system; 5 Sisal fiber bundles).

The continuous long sisal fiber/PLA premixes were prepared using an online extrusion-rolling blending system made in the laboratory, as shown in Figure 2. The PLA bar was first extruded by the extrusion equipment, the Brabender single screw extrusion module (PLASTI-CORDER, Germany). The heating temperature profile for the feeding zone, compression zone, metering zone and the die exit was set at 170, 180, 180 and 170 °C, respectively. It was found that when the screw speed was 2 r/min and the belt speed was more than 1.35 m/min, a stable mass flow rate of extruded PLA bar could be obtained. In this case, the PLA bar could maintain a certain degree of fluidity when it was extruded out of the die, and the temperature of the extrudates was not too high to adhere to the rubber belts.

While the PLA was stably extruded, the dried fiber bundles were introduced into the initial end of two conveyor belts through a fiber guide device to meet the extruded PLA bar. After that, the upper and lower conveyor belts pulled and rolled them to obtain continuous premixes of long sisal fiber and PLA. In order to achieve a better blending effect, as shown in Figure 2, the upper and lower conveyor belts were arranged at a particular angle (*θ* = 17°). As a result, the conveyor belts not only provided traction, but also had two different twisting effects (v_1_, v_2_, perpendicular to the extrusion direction) on the blends, as shown in the sectional view of Figure 2. Therefore, the fibers were twisted together with the extruded PLA bar. This allowed the PLA bar to be better coated or entangled with the fiber for better adherence. 

**Figure 3:**
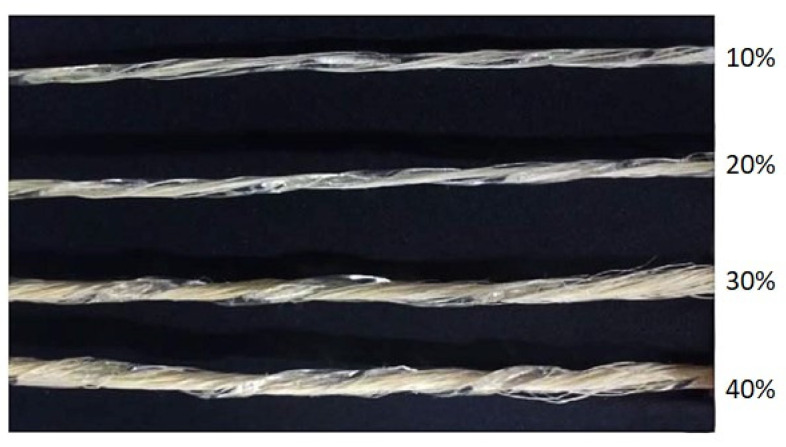
Continuous long sisal fiber/PLA premixes with different fiber loadings (10%, 20%, 30%, 40%).

The mass flow rate of PLA stayed the same after stable extrusion. On this condition, sisal fiber/PLA premixes with fiber loadings of 10%, 20%, 30% and 40% were prepared by introducing fiber bundles of different mass size, as shown in Figure 3. The liner mass of fiber bundles can be computed with the following formula:

$$m_{i}\;=\frac{m_{0}}{1-i}\;-\;m_{0}$$

where *m*_0_ and *m_i_* are the linear mass of stable extruded PLA bar and fiber bundles, respectively; *i* is the fiber loading (*i* = 10%, 20%, 30%, 40%). Finally, the continuous premixes were cut to 8–10 cm for a later molding process. It should be noted that this online extrusion-rolling premixing process could continuously premix long fiber reinforcements of unlimited length. The damage to fiber length and mechanical strength during this process was small, and the fiber could maintain a good orientation. Being an ecofriendly and practical technique, the extrusion-rolling melt blending method could be a feasible approach towards preparing continuous long fiber premixes.

#### 2.3.2. Preparation of Short Sisal Fiber/PLA Premixes 

The length of plant fiber is easy to decrease under severe mechanical shear in some commonly used fiber-reinforced biocomposite processing methods (like extrusion, injection and internal mixing) [29]. Therefore, for comparative analysis, mixtures of short sisal fiber and PLA were prepared using an open two-roller mill (SK-160B, China). PLA pellets dried at 80 °C for 3 h were melted in the open mill, and then sisal fibers were added for blending. The open mill had a front roll temperature of 185 °C, a rear roll of 180 °C and a roll speed of l0 r/min. To facilitate feeding, the sisal fibers were cut to ~10 mm prior to mixing. After blending for 5 min, the premixes were taken off and cooled down for later molding. The fiber loadings of short sisal fiber/PLA premixes were 10%, 20%, 30%, 40%, respectively. 

### 2.4. Molding of Composites

The premixes obtained by the two different blending methods were hot-pressed into composite sheets by a compression-molding machine (QLB-25D/Q, China). The molding temperature was set to 185 °C, and the pressure was set to 10 MPa. The premixes were first preheated in a mold for 10 min, then vented, and then were finally hot-pressed for 5 min at the set temperature and pressure. In addition, as to the long sisal fiber/PLA premixes, they were uniformly put in the mold in parallel to produce unidirectional long sisal fiber-reinforced PLA composites. Finally, a composite sheet was obtained by cooling the mold. 

**Figure 4:**
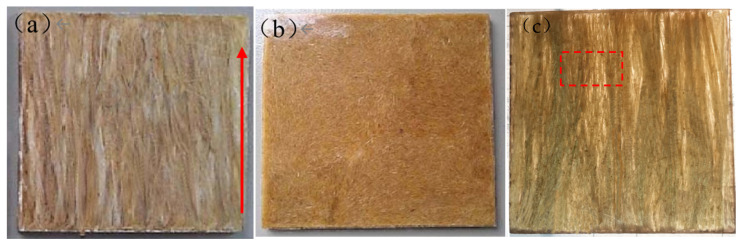
Comparison of two composite materials after molding (**a**) long sisal fiber-reinforced PLA composites [LSFCs], (**b**) short sisal fiber-reinforced PLA composites [SSFCs], (**c**) laminated composites).

For comparison, some fibers were cut short to have the same length as the hot-press mold. Then they were laminated into the mold with PLA pellets to make laminated SF/PLA composites with fiber loadings of 10% and 20%, respectively. Composites with fiber additions over 20% could not be molded well with direct hot-pressing because the feeding process was very difficult. Meanwhile it was not easy to control the fiber arrangement or dispersion in composites. Figure 4 is the comparison of long sisal fiber-reinforced PLA composites (LSFCs), short sisal fiber-reinforced PLA composites (SSFCs) and the laminated composites, where the red arrow represents the direction of fiber elongation. Premixes were molded into sheets with dimensions of 100 × 100 × 4 mm^3^ for flexural or impact tests and 160 × 80 × 1 mm^3^ for tensile tests, respectively. 

### 2.5. Composite Characteristics

#### 2.5.1. Mechanical Tests 

The tensile, flexural, and impact properties of the composites were tested to determine their mechanical behavior. The obtained composite sheets were cut into standard sample size, according to ISO527 for the tensile test, ISO178 for the flexural test and ISO180 for the notched Izod impact test. For LSFCs, the test samples were cut along the fiber orientation to retain long fibers. 

An electronic all-powerful experiment machine (Instron 5566) was employed to test the tensile properties and flexural properties of the composite samples. The tensile rate for all composite samples was 2 mm/min, and the loading speeding for flexural properties testing was 2 mm/min. A cylinder support beam impact-testing machine (Dynatup POE2000) was used to conduct the impact strength tests. All tests were performed with at least five samples at room temperature, and the corresponding properties were averaged. 

#### 2.5.2. Scanning Electron Microscopy 

The cryo-fractured surface microstructures of the compression molded sheets of SSFCs and LSFCs were studied using a Quanta 200 (FEI, USA) scanning electron microscope (SEM).

## 3. Results and Discussion

### 3.1. Tensile Tests

**Figure 5:**
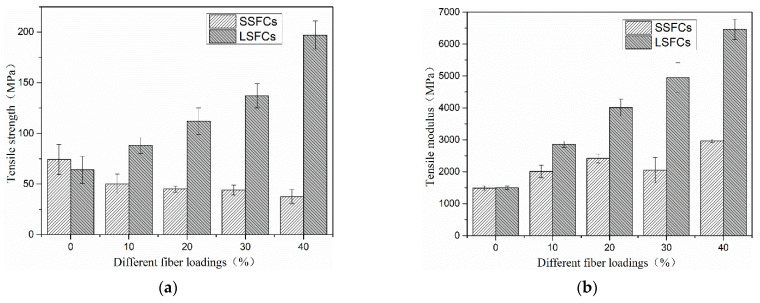

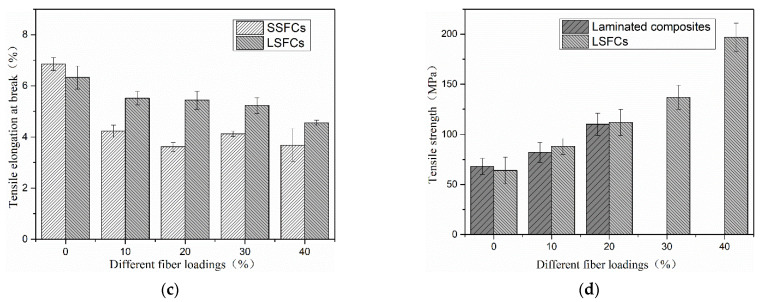
Tensile properties of composites: (**a**,**d**) tensile strength, (**b**) tensile modulus, (**c**) elongation at break.

The mechanical properties of composites are not only determined by the mechanical properties of the matrix and fiber themselves, but are also related to the content of the fiber, the length of the fiber, the orientation of the fiber and the strength of the interface between the fiber and the matrix. Figure 5 shows the tensile results of both composites. It can be seen from Figure 5a that the tensile strength of SSFCs reduced with the addition of short sisal fiber, compared with pure PLA. Sisal fibers show obvious hydrophilicity, while PLA molecules are hydrophobic [3]. The poor compatibility between PLA and sisal fibers makes it easy for defects to appear in the interfacial layer of the composite, such as voids [30], which greatly affects interfacial strength. Because of the shearing effect of the open mill during the blending process, the retain length of the sisal fiber was further shortened in SSFCs. Therefore, when the composite material was subjected to stretching, the stress on the matrix could not be transferred well to the fiber. In addition, the presence of more fiber ends in SSFCs was likely the cause of stress concentration and thus accelerated cracks in the composite material [9]. As fiber content increased, the probability of interfacial defects increased as well. 

However, the premixes of SF and PLA prepared by the extrusion-rolling premixing process had basically no mechanical damage, and the fiber still maintained its original length and mechanical strength in the matrix. During hot-press molding, the fibers were placed in the best orientation. Therefore, when the composite material was under tension, the fiber and the matrix could effectively transmit tensile stress. The presence of continuous long fibers could avoid defects caused by short fibers. Therefore, the strength of the composite material was mainly determined by the fiber and the matrix themselves. The tensile strength of the composite material increased along with the increase in fiber loading. The tensile strength reached a maximum of 200.44 MPa when the fiber content was 40 wt%, which was 3.06 times that of pure PLA. As can also be seen in Figure 5b, the continuity or orientation of fiber in the LSFCs made the tensile modulus increase rapidly as the fiber content increased.

Figure 5c is a graph of the elongation at break of different composites with different fiber loadings. It could be seen that the elongation at break of the composite materials obtained by the two blending methods was somewhat lower than that of pure PLA. It was a collaborative result of the brittleness of the PLA and the low elongation at break of the sisal fibers. The LSFC material had a smaller decrease in tensile elongation at break. This was due to the fact that in LSFCs, the fibers maintained their original length and good orientation. When the composites were stretched, the fiber and the PLA matrix exhibited different resistance to deformation. Attributed to the weaker interface binding between the fiber and the matrix, there was a relative slip between the two phases, which was a so-called fiber pull-out. Fiber pull-out is a process of overcoming friction to produce slip, which allows an increase in elongation at break of the composites. 

Figure 5d shows a partial comparison of the tensile strength of laminated composites and LSFCs. The laminated composites were directly prepared with long SF and PLA by hot-pressing. The tensile strength of the LSFC material increased with the addition of SF, and it was slightly higher than that of the laminated composites with the same fiber content. As for the laminated composite, it was difficult to mold by directly hot-pressing with high fiber loadings of more than 20%. For laminated composites, different lay-up methods need to be considered to ensure a uniformly mixed fiber and matrix [31]. This extrusion-rolling blending technology could prepare continuous long plant-fiber-reinforced premixes, and the fibers obtained a good dispersion in the matrix. 

### 3.2. Flexural Tests

**Figure 6:**
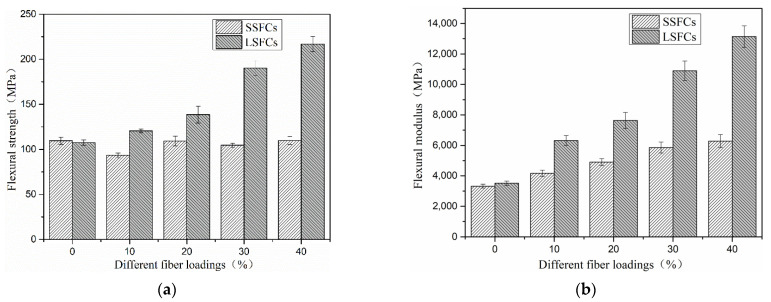
Flexural properties of composites: (**a**) flexural strength, (**b**) flexural modulus.

Figure 6 shows the curve of the flexural strength of the two composites as a function of fiber loadings. It can be seen from Figure 6 that the flexural performance of the SSFC materials had no obvious improvement compared with pure PLA. However, the LSFC materials shown a significant increase in flexural strength as the fiber content increased. The maximum flexural strength of the long fiber-reinforced composite material reached 216.77 MPa. The load-sharing capacity of the composites mainly depends both on the interface bonding between the fiber and the matrix, and on the orientation and dispersion of fibers [32,33]. Low interface bonding strength had a great influence on the mechanical properties of fiber-reinforced composites. Nonetheless, fiber continuity and orientation in the LSFC materials greatly reduced this effect. In the direction of the applied bending load, all fibers in fracture surface could participate in sharing stress on the matrix.

**Figure 7:**
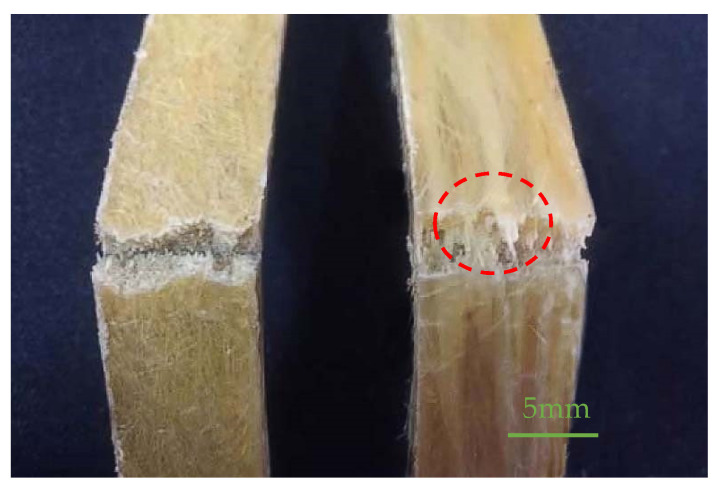
The flexural fracture diagram of composites (**left**: SSFCs, **right**: LSFCs).

The picture on Figure 7 shows fracture diagram of two different composite materials after bending failure. It is clear that the fiber broke when the composite cracked. The addition of fibers improved the flexural modulus of the composite material, as shown in Figure 6b, indicating that the stiffness of the resulting composite material increased.

### 3.3. Impact Strength

**Figure 8:**
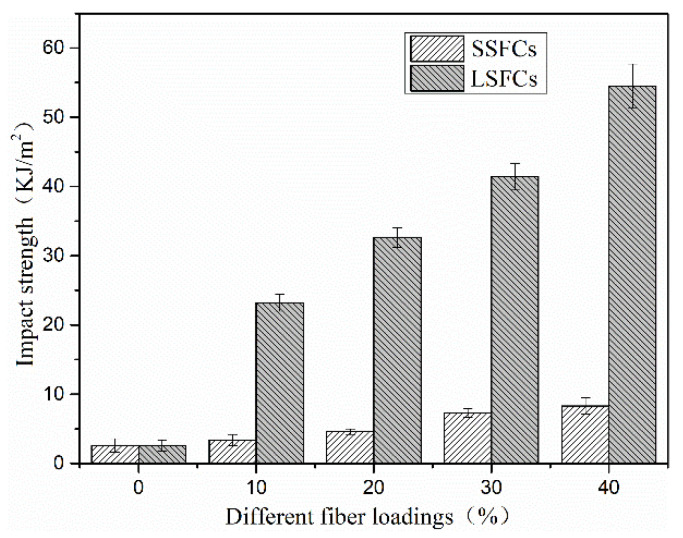
Impact strength of developed composites.

The notched impact strength of the two composite materials had been improved to a different degree compared to pure PLA, and both of them show a trend of increasing impact strength as their fiber content increased (Figure 8). Among them, SSFC material had a maximum impact strength of 8.216 KJ/m^2^ when the fiber content was 40 wt%, which was 2.62 times higher than that of pure PLA. Meanwhile, the impact strength of continuous long sisal fiber-reinforced composite material increased to 54.470 KJ/m^2^, which was 23.52 times that of pure PLA. There are three ways for fiber-reinforced composites to absorb impact energy [34]: matrix breakage, fiber breakage and fiber pull-out. PLA is a notch-sensitive material, and its impact strength is low. When the composite material is subjected to impact stress, the breakage or pull-out of the fiber can dissipate more energy, which correspondingly improves the impact strength of the composite material.

**Figure 9:**
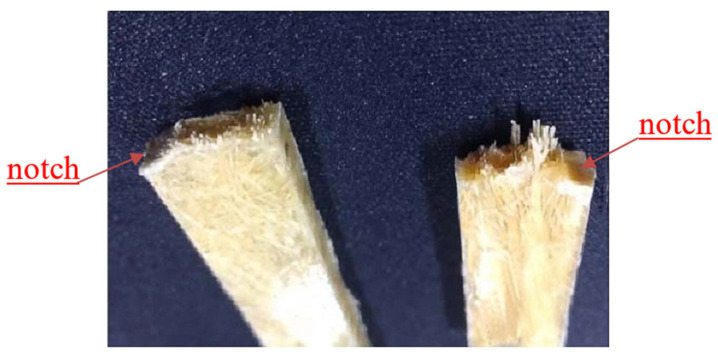
The impact fracture diagram of composites (**left**: SSFCs, **right**: LSFCs).

The energy absorbed in terms of fibers being pulled out of the matrix has been reported to be higher than that absorbed during fiber breakages [35,36]. Poor compatibility makes the interface bonding strength between the fiber and the matrix poor. When subjected to impact loads, cracks are often generated in the weakest interfacial layer on composites, which eventually leads to material fracture. In SSFC material, the fibers were short and dispersed randomly. Only a few fibers were broken or pulled out during the impact fracture, so the improvement in the notched impact performance of SSFCs was limited (Figure 9). In LSFCs, the fibers maintained their original length and were arranged in parallel. When subjected to an impact load, a large number of fibers were broken or partially pulled out, absorbing a large amount of energy, which thus improved the impact strength of the material greatly. As the fiber content increased, this advantage became more and more outstanding. Therefore, the extrusion-rolling premixing method could significantly improve the impact strength of LSFC material.

### 3.4. SEM Microstructure of Composites

**Figure 10:**
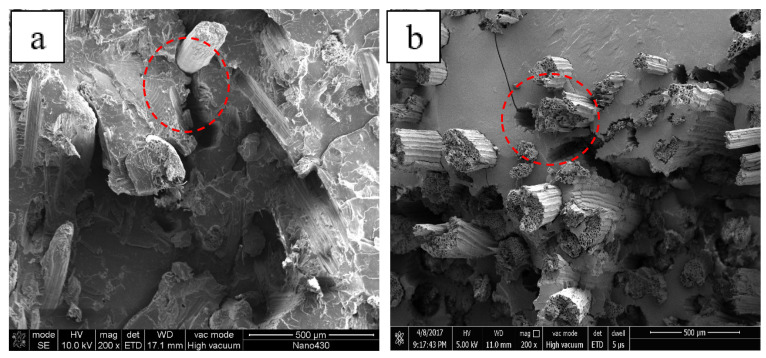
SEM micrographs of the cryo-fractured surfaces of the composites: (**a**) SSFCs and (**b**) LSFCs.

Figure 10 shows the micrographs of the fracture surfaces of two composites with a fiber loading of 20%. It can be seen that there were many holes and the fracture surface was quite uneven in SSFCs, which were caused by the debonding of the fibers from the matrix during the destruction of the composite. That implied only a few fibers were subjected to stress. Therefore, short fibers had a poor reinforcing effect on the composites. For LSFCs, though, the fracture surface was relatively neat and the PLA matrix had a good wrapping effect on the fibers. The fibers appeared partially pulled out during the fracture process. The long fibers were all involved in the force at fracture, which was also the reason for the obvious enhancing effect of the LSFC material. 

## 4. Conclusions

In this paper, two kinds of sisal fiber-reinforced polylactic acid biocomposites with different fiber lengths were prepared, including random short sisal fiber-reinforced PLA composites prepared by open milling and unidirectional long sisal fiber-reinforced PLA composites prepared by extrusion-rolling blending and molding. The effects of preparation technology and fiber length on the tensile, flexural and impact properties of composite materials were studied. 

The results show that, for LSFC material, the tensile and flexural strength or modulus improved with increases in fiber content. Due to poor compatibility between PLA and fiber, the addition of fibers made the tensile strength of the composite decrease in SSFCs. The elongation at break of both composites decreased with the addition of fiber loadings, but the decrease in LSFCs was smaller than that of SSFCs. The notched impact strength of LSFCs had been significantly improved compared with pure PLA and SSFCs. The extrusion-rolling blending process did little damage to the fiber length and its mechanical strength, and the fiber orientation in the composite material was controllable. Continuous long fibers could reduce the stress concentration problem caused by weaker interfacial performance between the two phases. Therefore, the mechanical strength and modulus of the composite material had been highly improved. Extrusion-rolling blending technology is a prospective method for plant-fiber-reinforced polymer composites manufacture.
